# Impact of Polyester Dendrimers as Branched Multifunctional Cross-Linking Additives in Triazine-Trione-Based Composites Developed via High-Energy Visible Light Thiol–ene Chemistry

**DOI:** 10.1021/acsapm.3c02246

**Published:** 2023-11-27

**Authors:** Jinjian Lin, Natalia Sanz del Olmo, Jorge San Jacinto Garcia, Faridah Namata, Daniel J. Hutchinson, Michael Malkoch

**Affiliations:** School of Engineering Sciences in Chemistry, Biotechnology and Health (CBH), Department of Fibre and Polymer Technology, KTH Royal Institute of Technology, Teknikringen 56-58, SE-100 44 Stockholm, Sweden

**Keywords:** triazine-trione materials, thiol−ene chemistry, high mechanical properties, degradability, dendrimers, viscosity, biocompatibility

## Abstract

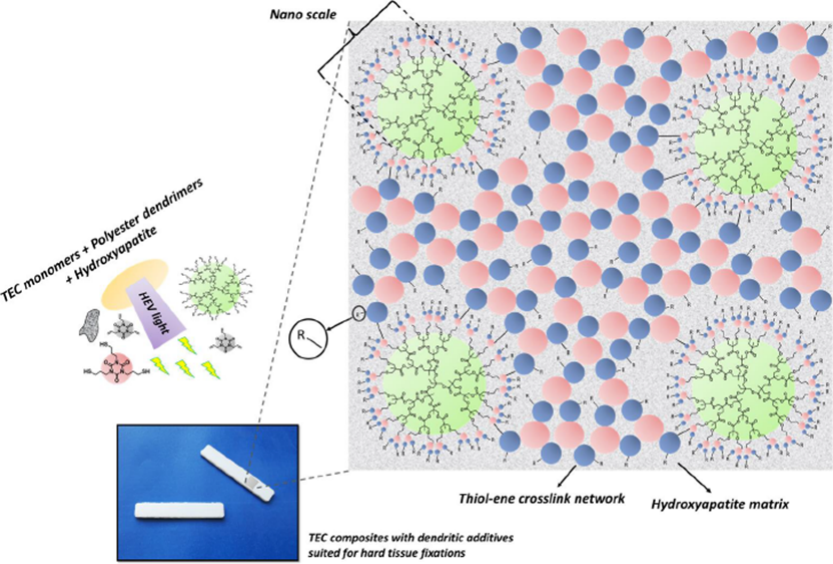

Hydroxyapatite (HA) infused triazine-trione (TATO) composites have emerged as an injectable platform for customizable bone fixators due to their fast and benign curing via high-energy visible light-induced thiol–ene chemistry (HEV-TEC), promising mechanical performance, and preclinical outcomes. These composites can overcome many of the existing limitations accompanying metal implants such as poor patient customizability, soft tissue adhesions, and stress shielding. Taking into account that the promising benchmarked TATO composite (BC) is based on stable sulfur–carbon bonds, we herein investigate the impact of introducing polyester dendritic cross-linkers based on bis-MPA as chemically integrated branched additives that display labile esters in a branched configuration. The inclusion of dendrimers, G1 and G3, in concentrations of 1, 3, and 5 wt % in the composite formulations were found to (i) decrease the processing viscosity of the composite formulations, reaching Newtonic and nonshear thinning behavior at 37 °C and (ii) impact the size distribution of bubble cavities in the composite cross sections. The lowest collected *T*_g_ for the dendrimer-containing composites was noted to be 73.2 °C, a temperature well above physiological temperature. Additionally, all composites displayed flexural modulus above 6 GPa and flexural strength of ca. 50 MPa under dry conditions. The composites comprising 5 wt % of G1 and G3 dendrimers, with ester bond densities of 0.208 and 0.297 mmol/g, respectively, reached a mass loss up to 0.27% in phosphate buffered saline at 37 °C, which is within the range of established polycaprolactone (PCL). Combined with the nontoxic properties extracted from the cell viability study, polyester dendrimers were determined as promising additives which compatibilized well with the TATO formulation and cross-linked efficiently resulting in strong composites suited for bone fracture fixations.

## 1 Introduction

Bone fractures are serious injuries that affect millions of people globally and cause a heavy societal and economic burden.^1,2^ Depending on the severity of the fracture, conservative methods, such as casts and splints, or surgical methods, such as open reduction internal fixation (ORIF) plates and screws, should be applied to facilitate bone healing.^3−9^ ORIF is an invasive method that uses metal implants to provide mechanical support to unstable fractures. While ORIF implants provide exceptional stability to the healing bone, they come with disadvantages such as low customizability, stress shielding, and a tendency to cause adhesions to the surrounding soft tissue. This latter issue often results in a loss of mobility in nearby joints after healing, sometimes necessitating the removal of the metal implants in a second surgery.^10−14^

In order to overcome the issues associated with ORIF implants, our research group has developed a polymer composite-based bone fixation approach which allows the surgeon to create patient-customized composite fixators, with and without the use of screws.^15,16^ Osteosynthesis is achieved by sculpting a “plate” over the fracture in situ with layers of a fluid mixture, which is then cured in seconds via high energy visible (HEV) light-induced thiol–ene coupling (TEC) chemistry. Traditional metal screws are used to anchor the composite plate to the bone.^16^ The composite consists of trifunctional triazine-trione (TATO) alkene and thiol monomers as well as a high concentration (56 wt %) of hydroxyapatite microparticles (HA). Once cured, the nondegradable composite displays a high rigidity and strength, with a flexural modulus of 7.5 GPa.^16^ High flexural modulus is a crucial parameter for materials that are intended to stabilize fractures during the bone healing process. For the TATO systems, this was achieved by the introduction of HA microparticles as reinforcing fillers. For instance, the flexural modulus of TATO thermosets was noted to reach 2.8 GPa, while the addition of 56 wt % of physically entrapped HA yielded TATO composites with a flexural modulus of 6.6 GPa: an increase of 236%.^16^ The high surgical customizability of these composites emanates from their processable viscosity and the robustness of the TEC reaction, which is nonsensitive to oxygen inhibition, highly regioselective, cures rapidly under mild conditions, and has a high monomer conversion. Moreover, the strong and biocompatible TATO composite based on sulfur-ether bonds has a glass transition temperature (*T*_g_) of 77 °C after maximum water absorption and has been verified in preclinical studies to supply adequate mechanical support toward bone tissues without causing adverse effects, such as adhesions, on the surrounding tissues.^15−22^

The TATO-based composites fulfill many of the demands of a biomaterial intended for bone fixation; however, their applicability could be greatly expanded by altering the physicochemical properties of the composites. In this context, the introduction of monodisperse dendrimers as discrete multifunctional additives provides an avenue for structure-to-property correlation of the TATO-based composites.^23−27^ Dendrimers are three-dimensional, highly branched, and multifunctional scaffolds that were envisaged as promising additives to be covalently incorporated into the TATO composite networks. Aliphatic dendrimers with external allyl groups and internal ester linkages were considered as a logical next step that can potentially affect the formulation parameters as well as the overall properties of the cross-linked networks. More specifically, polyester dendrimers based on 2,2-bis(methylol)propionic acid (bis-MPA) were targeted as nanoscopic hydrolyzable nodes that can potentially promote hydrolytic or enzymatic degradation of the sulfur-ether TATO composites in physiological mimicking conditions.^28,29^ The bis-MPA dendrimer platform is well recognized for its chemical versatility including (i) the introduction of an exact number of functional groups in one single platform; (ii) compatibility with click reactions including TEC chemistry and (iii) a wide variety of monomers with different functional groups.^29^ Additionally, they have been widely researched for various uses in biomedical applications such as nanocarriers for the delivery of therapeutically active drugs and siRNA as well as antibacterial agents.^29−32^ Attractive features that accompany the utilization of bis-MPA based dendritic materials for life science applications are their commercial availability, high biocompatibility, and degradability. With respect to HEV-TEC compatibility, bis-MPA dendrimers displaying 3-(allyloxy)-2-((allyloxy)methyl)-2-methylpropanoic acid (BAPA) as the outer generation have been synthesized and adequately evaluated in the context of TEC click chemistry and antibacterial synthetic agents.^33^ The inherent capability of the BAPA-decorated bis-MPA dendrimers makes them ideal candidates for evaluation as precision multifunctional additives in thermoset and composite formulations with an emphasis on tissue fixation and regenerative applications.^34,35^

**Figure 1:**
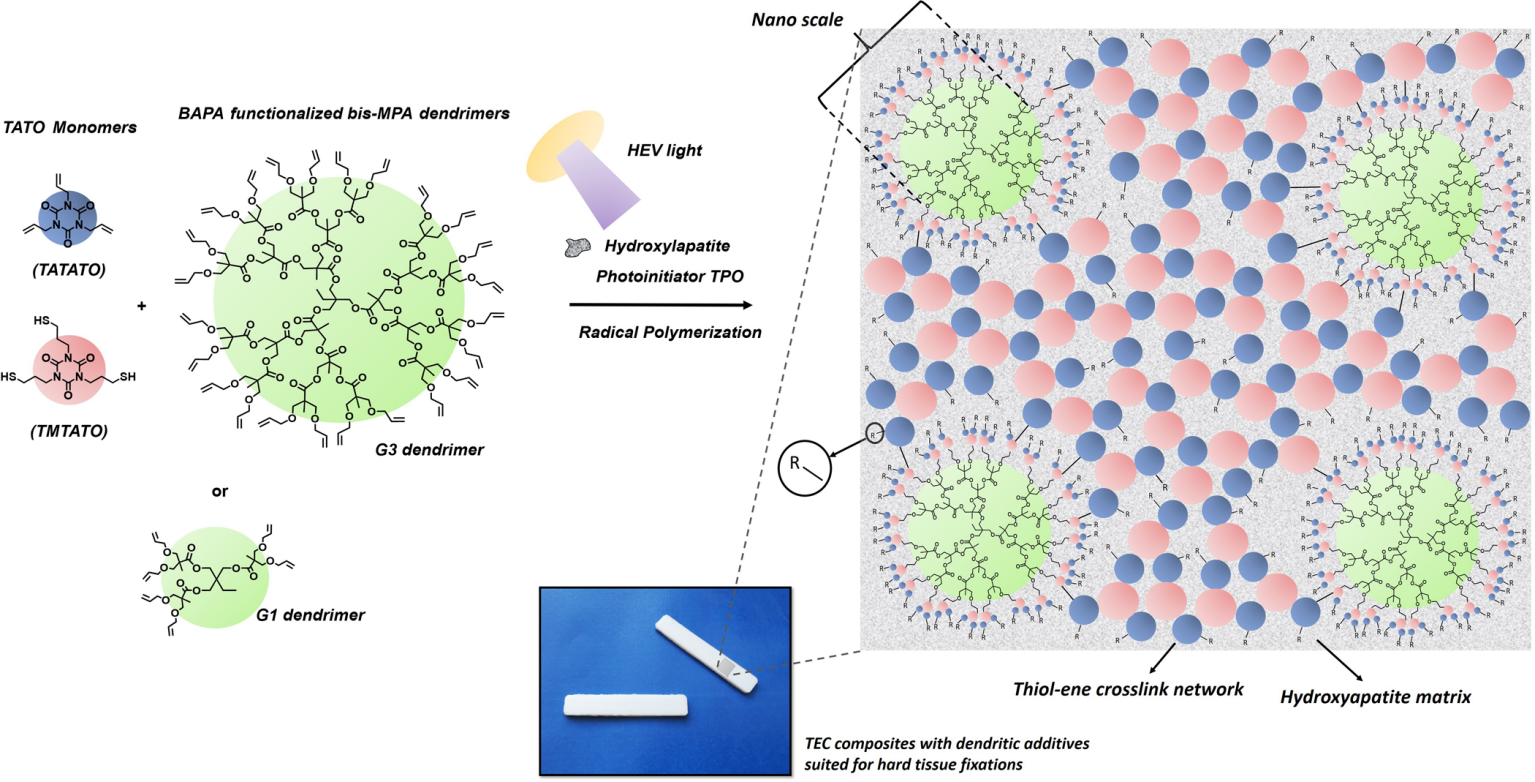
TATATO, TMTATO, and HA microparticles included in the BC formulation as well as the introduction of low wt % of G1 and G3 BAPA-decorated bis-MPA dendrimers. Formulations containing HA (56 wt %) together with TATATO, TMTATO, dendrimers, initiator, and catalyst (44 wt %) undergo efficient HEV-TEC curing to cross-linked composites in 10 s. “–R” group stands for possible extended cross-linked network.

Herein, we report the successful formulation and development of a TATO composite based on 1,3,5-triallyl-1,3,5-triazine-2,4,6-trione (TATATO) and 1,3,5-triazine-2,4,6-trione, 1,3,5-tris(mercaptopropyl) (TMTATO) monomers, together with HA and BAPA dendrimers as multifunctional dendritic additives. Dendrimers of the first and third generation (G) with 6 and 24 allyl groups, respectively, were introduced in 1, 3, or 5 wt % and formulated together with the TATO monomers at an equimolar ratio prior to cross-linking (Figure 1). The impact of the dendrimers on the formulation as well as the mechanical performance, degradation, and cytotoxicity of the composites was evaluated.

## 2 Experimental Section

### 2.1 Preparation of Materials

Materials TATATO, diphenyl(2,4,6-trimethylbenzoyl) phosphine oxide (TPO), and HA were purchased from Sigma-Aldrich Sweden AB. TMTATO was synthesized from TATATO according to a previous reported method.^36^ Cat57 was synthesized in-house.^37^ Trimethylolpropane (TMP) and 2,2-bis(methylol)propionic acid (bis-MPA) were generously provided by Perstorp AB, Sweden. Generations 1 and 3 bis-MPA dendrimers functionalized with an outer layer of BAPA, TMP-G1-BAPA_3_ (G1), and TMP-G3-BAPA_12_ (G3), were synthesized based on a previously reported protocol.^33^

Beams of the composites were constructed for three-point bending tests from rectangular molds (length 40 mm, width 6.5 mm, thickness 1.5 mm) produced from a Teflon sheet (RS components, Gothenburg, Sweden). Human epidermal keratinocyte (HaCaT) and mouse monocyte (Raw 264.7) cells were purchased from the American Tissue Culture Collection (ATCC). For the tests of cell viability, Dulbecco’s Modified Eagle Medium (DMEM), fetal bovine serum (FBS), and the mixture of antibiotics penicillin/streptomycin were purchased from Thermo Fisher Scientific.

### 2.2 Formulation and Curing of Composite Materials

Composites were made with TATATO, TMTATO, Cat57, TPO, and HA with either 0, 1, 3, or 5 wt % of G1 or G3. The different composite formulations are provided in the Supporting Information, Table S1. The procedures are given as described: TATATO, TMTATO, Cat57, TPO, and the dendrimer were mixed together in a vial while being protected from light. After a homogeneous mixture was obtained through stirring, HA was added and the mixture was stirred again until a homogeneous, white, viscous consistency was achieved.

Beams and discs of the composites were made for the purposes of mechanical analysis and water absorption by curing the composite in Teflon molds of dimensions 40 × 6.5 × 1.5 mm (length × width × thickness) or 12 × 1.5 mm (diameter x thickness), respectively. Curing was achieved with a portable high-performance LED lamp (Bluephase 20i, Ivoclar Vivadent AG, Leichtenstein) with a spectrum wavelength of 385–515 nm and a light intensity of 2000 mW/cm^2^. At least two pulses (5 s/pulse) of the LED treatment were applied to each surface area (a square of 1 cm^2^) on both sides of the composite surface to ensure full conversion of monomer.

### 2.3 Raman Spectroscopy

Monomer and dendrimer conversion upon curing was confirmed through the use of a portable i-Raman Plus spectrometer (model: BWS465-785S, B&W TEK). The surfaces of both the uncured resins and composites were analyzed with a total number of 48 scans (laser wavelength: 785 nm, laser power: 100%, integration time: 1000 ms). The raw data were then analyzed on BWSpec software and plotted on Origin 2020 (academy). The Raman intensity for the carbonyl functionality at 1760 cm^–1^ was used to normalize the spectra. The intensities of thiol groups (2575 cm^–1^) and C–C double bonds (1645 cm^–1^) were compared between the resins and cured materials to investigate the monomer conversion of TEC reactions.

### 2.4 Viscosity Measurements

The rheological properties of the uncured resins of the composites made with or without 5 wt % of G1 or G3 dendrimers were investigated with a DHR-2 rheometer from TA Instruments. A parallel geometry with a diameter of 20 mm and a geometry gap of 300 μm was applied for the measurements. Flow-sweep mode was selected to measure the relationships between the viscosity and the logarithm shear rate. The shear rate was set to increase from 10^–4^ to 10^2^ 1/s (5 data points per decade) during the test. The resins were tested first at 25 °C and then 37 °C and at least three different batches were tested for each resin at each of the two temperatures. The raw data were collected in log scales (base 10) and analyzed on Trios software (TA Instruments). All results are shown as mean (SD) after confirming no major deviation from three obtained viscosity-shear rate curves for each material at both 25 and 37 °C (Supporting Information, Figure S3).

### 2.5 Three-Point Bending Testing

All samples were tested under dry conditions, while only the composite without dendrimers and the composites containing 5 wt % of either G1 or G3 were tested under wet conditions after being immersed in PBS (pH 7.4) for 7 days. The dry condition tests were conducted 1 day after the sample beams were prepared. Meanwhile, for the wet condition tests, the samples were taken out from the PBS solution and dried with tissue paper to remove the excess water on the surface. The samples were then allowed to cool down to room temperature before three-point bending testing. Both dry and wet samples were tested with an Instron 5944 single column universal testing machine (Instron Korea LLC) with a load cell of 500 N, a cross-head speed of 1 mm/min, a preload of 0.1 N, and a preload speed of 0.5 mm/min. The center-to-center distance of the lower contacts was set to 30 mm and all measurements were conducted at 20 °C with a relative humidity of 50%. The data were analyzed and collected by Bluehill software. The flexural modulus was calculated by eq 1, where *L* is the lower contacts’ distance, *m* is the slope at the initial elastic region of the load and displacement curve, *w* is the width of the beam, and *d* is the thickness of the beam. For each material, at least five samples were tested, and the results are given as mean (SD):
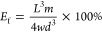
1

### 2.6 Dynamic Mechanical Analysis

The *T*_g_ of the BC and all of the dendrimer-modified composites were measured by a Dynamic Mechanical Analyzer (DMA Q800, T.A. Instruments, USA) in thin film/tensile mode. The dimensions of the materials were 12 × 6.5 × 1.5 (length x width x thickness). The samples were tested under both dry and wet conditions. A temperature ramp method with a heating rate of 3 °C/min was used and the testing temperatures ranged from either −10, −5, and 0 to 110, 120, and 130 °C, depending on the materials’ properties. A strain of 0.1% was induced with a frequency of 1 Hz. For each material at least five samples were tested. All results are shown as mean (SD).

### 2.7 Scanning Electron Microscopy (SEM) on Cross-Sections

The cross sections of the beams of the cured composites were observed and recorded by an FE-SEM S-4800 (Hitachi, Japan). The natural cross sections for characterization of all of the materials were cryo-fractured by liquid nitrogen for 5 min and were then fractured in the middle of the beams. The samples were coated with Au–Pt thin layers, making use of a Sputter-coater-JFC1300 (JEOL USA) and were then investigated by FE-SEM under 15 kV acceleration voltage. For each formulation, three specimens were observed under FE-SEM. Pore analysis of the SEM images was analyzed by the software ImageJ (NIH). A square area of 1.5 × 1.5 mm^2^ was selected from each SEM image and the histograms of the average pore sizes and counts were collected by filtering the pores with a size lower than 3 × 10^–4^ mm^2^.

### 2.8 Water Absorption and Degradability

The water absorption and degradability tests were conducted on the BC composite and all the composites containing 5 wt % G1 and G3. Five samples of each composite were prepared and cured in a plastic mold with dimensions of 12 × 1.5 mm (diameter x thickness). Thereafter, the samples were dried in a 50 °C oven until they obtained constant dried weights (*m*_1_) at which the dry weight differences stabilized within 0.1 mg. Afterward, they were stored separately in 30 mL vials filled with PBS solution (pH 7.4) in a 37 °C oven under different time points: 7, 14, 28, and 42 days for water absorption and 56 days for degradability. For the time points for water absorption, the samples were taken out from the oven and washed with deionized water, and then excess water was removed from the surface of the discs. Their wet weights (*m*_2_) were then recorded before they were returned to the PBS-filled vials in the 37 °C oven. The same procedure was repeated until the last time point of 42 days. Furthermore, on day 56 the samples were taken out from the vials and then washed completely with deionized water to remove possible salt residues on the surface of the discs. Afterward, they were dried in a 50 °C oven until they obtained a constant dried weight (*m*_3_). The water absorption (*M*_absorp_) and the degradability (*M*_deg_) of BC, 5 wt % G1 and 5 wt % G3 composites were then calculated, respectively, by eqs 2 and 3 and the absolute values were recorded and given as mean (SD):
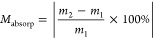
2
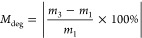
3

### 2.9 Cytotoxicity Assay

A monolayer of HaCaT and Raw 264.7 cells was used for the cytotoxicity tests. These cell lines were maintained in tissue culture flasks at 37 °C in 5% CO_2_ with Dulbecco’s Modified Eagle Medium (DMEM), supplemented with 10% (v/v) fetal bovine serum, l-glutamine (4 mM), 100 IU mL^–1^ penicillin, and 100 μg mL^–1^ streptomycin. A solution of 100 μL of the cells at a concentration of 1 × 10^4^ cells per well was harvested in 96 well plates for the cytotoxicity test and incubated for 24 h at 37 °C and 5% CO_2_. Meanwhile, the composites were exposed to UV light for 20 min. Afterward, discs of the cured composites, with dimensions of 12 × 1.5 mm (diameter x thickness), were transferred into 2 mL of complete DMEM and incubated at 37 °C for 24 h to get the leach-out medium (the testing medium). Afterward, the old cell culture medium was replaced by 100 μL of the testing medium per well and incubated for 72 h. For each sample medium, six parallel wells were used, and three discs were used for each composite. Cells without treatment were used as a control, and extracts without cells were used as the blank of this experiment. Then, 10 μL of AlamarBlue agent was added to the cells and incubated for 4 h at 37 °C in 5% CO_2_. Finally, fluorescence intensity was measured with a plate reader (Tecan Infinite M200 Pro) at wavelengths of 560/590 nm (excitation/emission). All results are shown as mean (SD).

## 3 Results and Discussion

In order to fulfill their role as degradable additives in the TATO composite formulations, the allyl-functionalized bis-MPA dendrimers were designed with the following important physiochemical properties in mind: (i) the incorporation of ester-bonds into the polymer network which should function as degradable nodes, (ii) the presence of an exact number of allyls capable of forming chemical bridges with the TATATO and TMTATO monomers, and (iii) branched configuration that does not largely affect the viscosity or processability of the composite during the formulation. The chosen dendrimers had a bis-MPA architecture with a TMP core and an outer generation with allyl functional groups. Generations 1 and 3 were used as additives in order to investigate the impact of the dendrimer size and the concentration of allyl groups on the formulation and properties of the composites. The first-generation dendrimer (G1) comprising six allyls and three ester bonds represents a compact oligomeric cross-linker while the third-generation dendrimer (G3) is characterized as a highly branched polymeric additive with an exact number of 24 peripheral allyl groups and 21 ester-bonds reaching a molecular weight of 3534 g/mol. Both dendrimers were synthesized via a divergent growth approach^33^ and formulated together with TATATO, TMTATO, and HA fillers in a stochiometric ratio between the −SH and −C=C groups. Following earlier optimized conditions for TATO composites based on TEC-HEV chemistry, all mixtures contained HA, as a modulus enhancer, in a fixed concentration of 56 wt %. Additionally, the dendrimers were included in 1, 3, and 5 wt % in the formulations (Supporting Information, Table S1). The highest wt % took into consideration the flexible nature of the aliphatic dendritic skeleton and as a means to maintain sufficient mechanical properties of the TATO composite systems to potentially be utilized for hard-tissue fixations. As a guiding principle of sufficient mechanical support, commercial dental resin composites have a typical flexural modulus (*E*_f_) that ranges between 5 and 7 GPa.^38,39^

**Figure 2:**
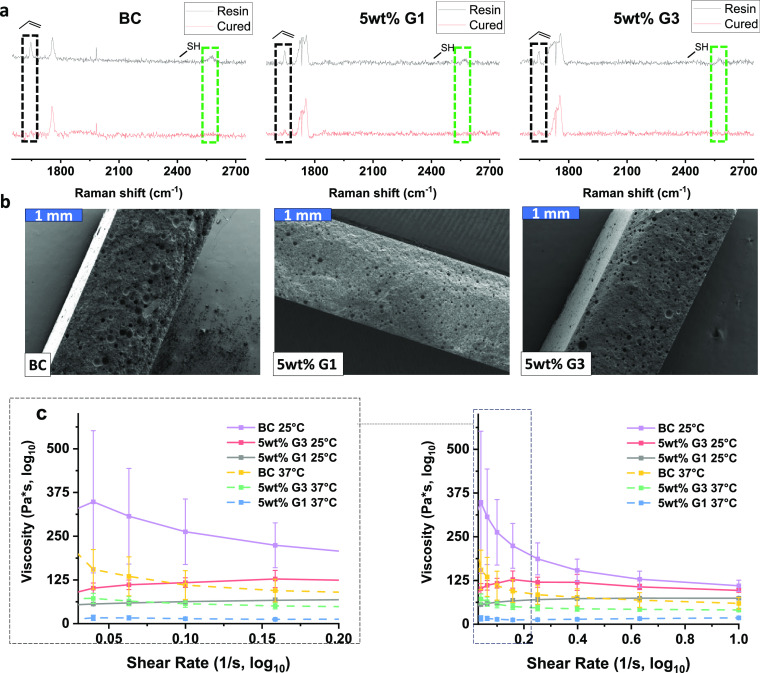
(a) Raman spectra before and after HEV light curing of the BC resins as well as with 5 wt % of G1 and G3 allyl-functional dendrimers (black dashed box for C=C bonds and green dash box for −SH bonds); (b) cross-sectional images of BC, 5 wt % G1 and 5 wt % G3 composites captured by FE-SEM; (c) rheological assessment of viscosity for the BC and 5 wt % of dendrimers included composites at both 25 and 37 °C.

Initially, cross-linking efficiency via HEV-TEC of the stoichiometric formulations with 1 or 5 wt % of the dendrimers was performed and compared to the neat composite system. Rectangular beams were produced using wavelengths of 385–515 nm and at a light intensity of 2000 mW/cm^2^. The formulations were cured using two five-second pulses and the final beams were examined using FT-RAMAN. The curing via HEV-TEV and the consumption of thiols and alkenes, with and without dendritic additives, followed the same efficiency (Figures 2a and S1). These findings indicated that the aliphatic thiols in the TMTATO monomer had the capacity to react with the allyl groups on both TATATO and the dendrimers, despite them being connected via carbon to either a nitrogen or oxygen atom, respectively.

The viscosity of the mixtures prior to cross-linking is an important rheological processing step that is dependent on the temperature, the composition of the mixtures, and the interactions between all components. All components in this study are considered hydrophobic and upon mixing homogeneous solutions were obtained. Considering their highly branched structures, the introduction of dendrimers to the mixtures was postulated to provide a shear-thinning effect to the viscosity. A series of studies of the viscosity versus shear rate at 25 and 37 °C revealed that the BC composite has shear thinning and non-Newtonian behavior at both temperatures and across the shear rates of 10^–4^–10^2^ 1/s (Figures 2c and S3). Analysis of the 5 wt % G1 and G3 composites showed that the addition of the dendrimers had a dramatic impact on the viscosity, with a less pronounced Newtonic behavior for the 5 wt % G1 at 25 and 37 °C as well as for the 5 wt % G3 at 37 °C (Figures 2c and S3). In general, the viscosity was lower at higher temperatures within the same system. Stable values of viscosity, at the lowest shear rate of 0.398 1/s, were measured as 154.0 (32.0) Pa s for the BC, 120.0 (23.0) Pa s for 5 wt % G3 and 73.1 (3.8) Pa s for 5 wt % G1 composites. At a similar shear rate and temperature of 37 °C, the viscosity decreased within each respective system to 76.8 (24.4) Pa s for the BC, 43.9 (7.0) Pa s for 5 wt % G3 and 14.1 (2.2) Pa s for 5 wt % G1 composites. Interestingly, at 5 wt % the G1 dendrimer with 6 allyls represented in a dendritic configuration resulted in a nearly nonshear thinning Newtonian behavior across the shear rate scale of 10^–4^–10^2^ 1/s. Based on these results, dendrimers may facilitate future formulations with increased filler content or other mechanical enhancing components while maintaining the desired viscosity suited for surgical fixation of fractures.

**Figure 3:**
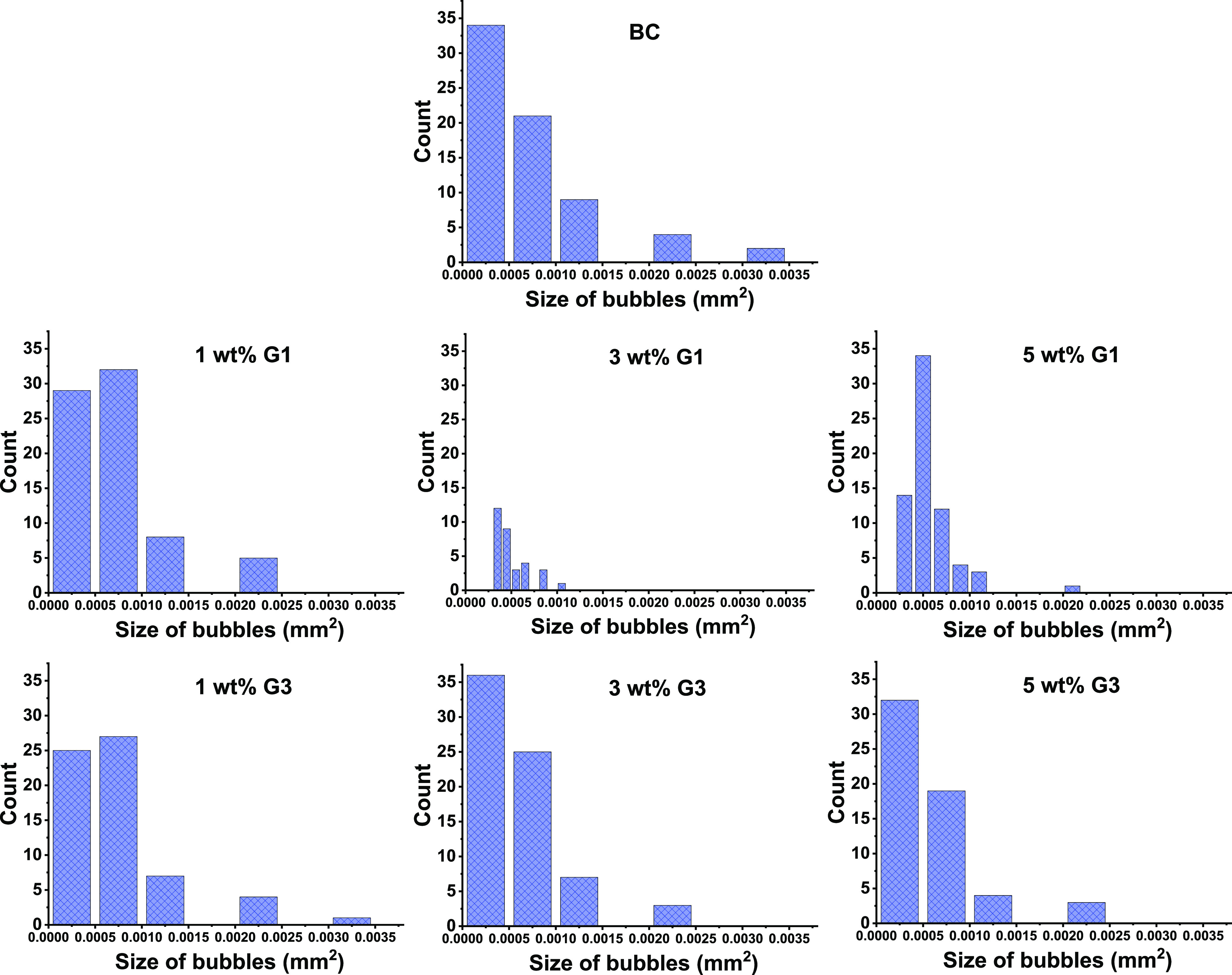
Histograms of FE-SEM analysis of size distribution and average size of pores within the composite area of 1.5 × 1.5 mm^2^. The size threshold is 0.0003 mm^2^ – infinity.

FE-SEM imaging of the composite cross sections revealed that all composites displayed the presence of micrometer cavities after HEV light curing, which were correlated to entrapped air bubbles generated during the mixing procedure (Figures 2b and S2). Visual examination of the composites with dendrimers as additives showcased similar macroscopic features to the composite without dendrimers. The cross sections were further analyzed with respect to size distribution and number of pores in the final composites (Figure 3). Interestingly, histogram results showed that the size distributions of the 1 wt % G1 and all G3 included composites were similar to the BC. The composites with 3 and 5 wt % G1 displayed a narrower size distribution when compared with the other composites. As seen in Figure 3, the majority of cavities in the composites with 3 and 5 wt % G1 were smaller than 0.00125 mm^2^, while sizes larger than 0.00200 mm^2^ could be seen in BC, 1 wt % G1 and all the G3 included composites.

To investigate the effect of the dendrimers on the composites’ *T*_g_, DMA was performed (Table 1). The addition of dendrimers resulted in an overall decrease in *T*_g_ measured in dry conditions, from 89.3 (1.2) °C for the BC to 77.4 (0.4) and 81.8 (1.5) °C for 5 wt % G1 and G3, respectively. In both systems, the lowest *T*_g_ was observed for composites containing 3 wt % of dendrimers with values of 74.8 (1.1) °C for G1 and 73.2 (0.8) °C for G3. The composites with the highest wt % of dendrimers and ester-bond density were further assessed under wet conditions. For BC, the *T*_g_ decreased by 8 °C to a value of 81.5 (0.6) °C. A similar decrease was observed for the composites with 5 wt % G1 and G3 decreasing in wet conditions to 68.3 (1.2) and 70.3 (0.8) °C, respectively. These *T*_g_ values, in both dry and wet conditions, were adequate with respect to maintaining glassy properties in physiological environments, which is an important consideration for the use of these composites in bone fracture fixations.

**Table 1:** Properties of Different Composites Based on the BC (**thiol** × **alkene** × **HA**) and Composites Containing Dendrimers from the First to Third Generation at Different Weight Percentages (1, 3, and 5 wt %) as Additives with the Results Representing the Average and the Standard Deviation of at least Five Repeats

materials	ester density (mmol/g)	*E*_f_ (dry) [MPa]	max σ_f_ (dry) [Mpa]	*T*_g_ (dry) [°C]	*E*_f_ (wet) [Mpa]	max σ_f_ (wet) [Mpa]	*T*_g_ (wet) [°C]	η (γ̇ of 0.398 1/s, 25 °C) (Pa s)	η (γ̇ of 0.398 1/s, 37 °C) (Pa s)
BC	0	7015 (157)	61 (1)	89.3 (1.2)	5881 (89)	40 (1)	81.5 (0.6)	154.0 (32.0)	76.8 (24.4)
1 wt % G1	0.041	6220 (119)	47 (2)	79.4 (0.6)					
1 wt % G3	0.059	6333 (116)	49 (1)	80.3 (0.7)					
3 wt % G1	0.125	6128 (112)	49 (2)	74.8 (1.1)					
3 wt % G3	0.178	6033 (191)	45 (2)	73.2 (0.8)					
5 wt % G1	0.208	6464 (109)	51 (2)	77.4 (0.4)	4861 (184)	37 (1)	68.3 (1.2)	73.1 (3.8)	14.1 (2.2)
5 wt % G3	0.297	6154 (100)	49 (2)	81.8 (1.5)	5476 (152)	36 (0)	70.3 (0.8)	119.6 (23.0)	43.9 (7.0)

**Figure 4:**
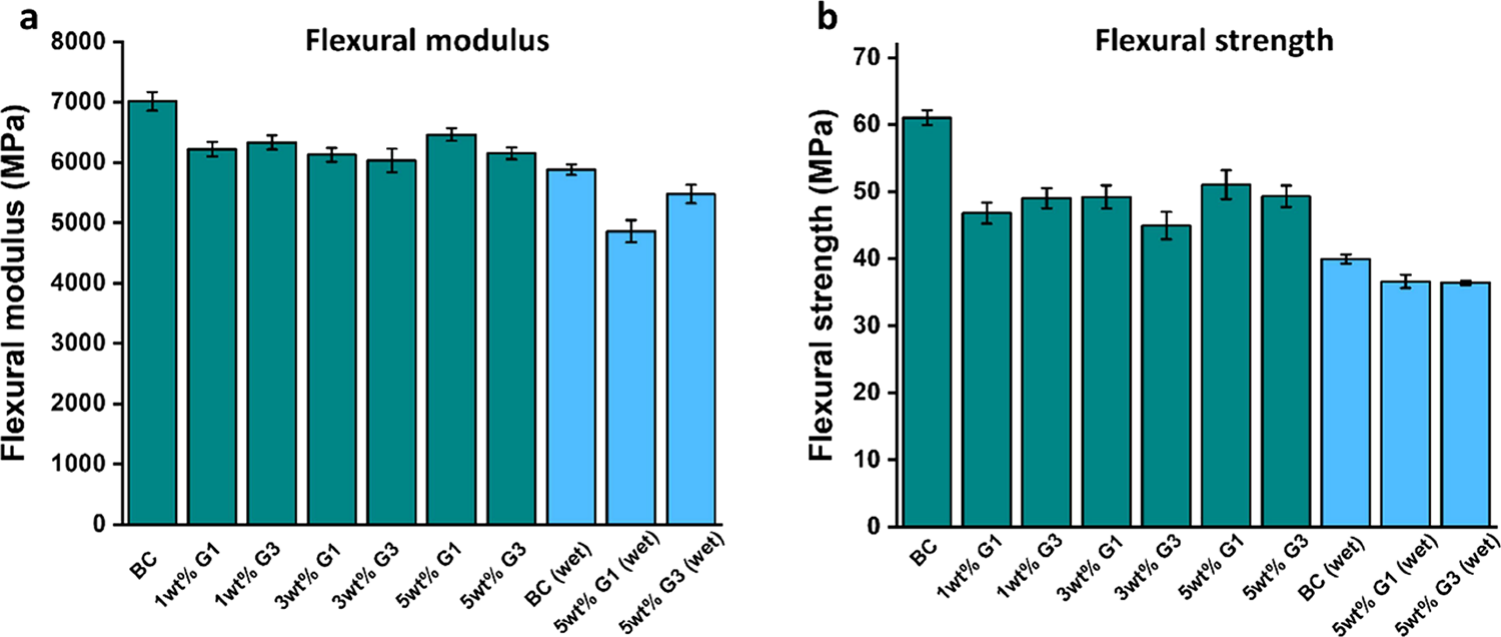
Collected (a) flexural modulus and (b) flexural strength for the BC and dendrimers included composites in dry (green) and wet (blue) conditions.

In terms of mechanical performance, three-point bending showed that the BC had the highest flexural modulus (E_f_) and flexural strength (σ_f_) of 7015 (157) and 61 (1) MPa, respectively (Table 1 and Figure 4). The inclusion of the G1 and G3 dendrimers resulted in a small decrease in modulus and strength; however, for all the composites these values were above 6 GPa and around 50 MPa, respectively. The lowest mechanical performance was noted for the composites with 3 wt % of dendrimers, which followed a similar trend for the *T*_g_ values. For the composites containing G1 dendrimers, as a small and ester-based cross-linking additive, the modulus reached the highest values at 5 wt % with a value of 6464 (109) MPa and flexural strength of 51 (2) MPa. This is in contrast to the composites with the largest dendritic additive, the G3 dendrimer, where the highest modulus of 6.2 GPa was found at 1 wt %. After water absorption, the mechanical performance of all of the composites decreased. The flexural modulus and strength of the BC composite after water absorption were 5.9 and 40 MPa, respectively, representing drops of 18 and 34% due to water absorption. The 5 wt % G1 composite after water absorption had a modulus of 4.9 GPa and a strength of 37 MPa, which corresponded to decreases of 25 and 27%, respectively. The decreases in modulus and strength were less severe in the 5 wt % G3 composite, which had a modulus and strength after water absorption of 5.5 GPa and 36 MPa (a decrease of 11 and 26%, respectively). This was surprising as the 5 wt % G3 composite had the highest density of ester-bonds, 0.297 mmol/g.

**Figure 5:**
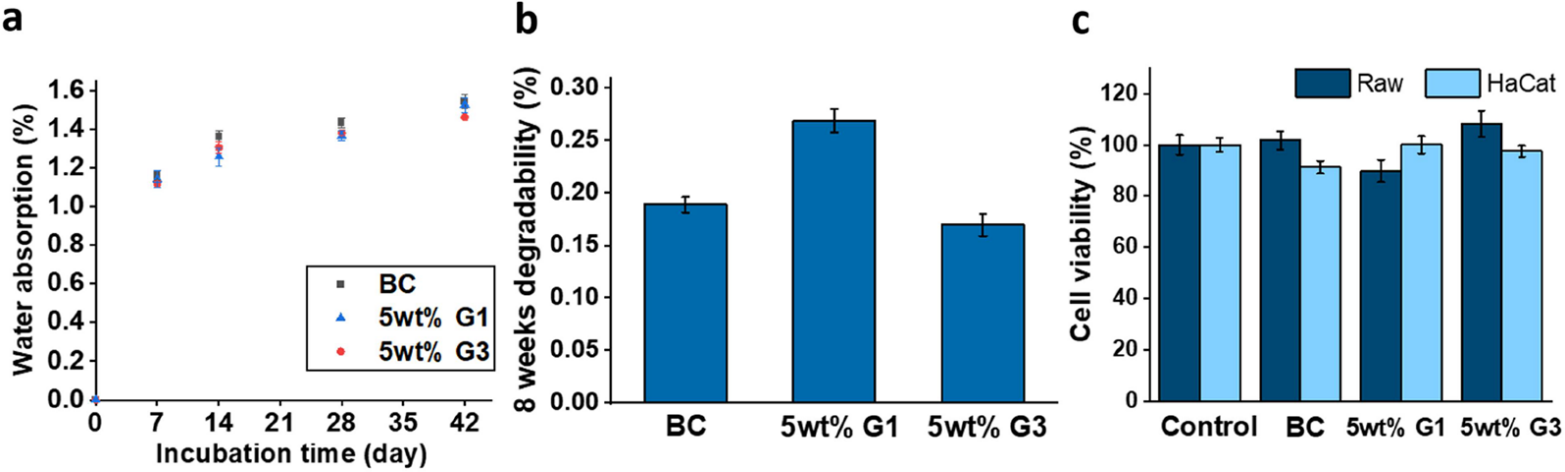
(a) Water absorption over a period of 42 days, (b) degradability over a period of 56 days, and (c) cell viability toward Raw and HaCaT cells of the BC and 5 wt % G1 and G3 composites.

The impact of the polyester dendrimers on the water absorption and degradation of the composites was investigated by soaking the BC, 5 wt % G1, and 5 wt % G3 composites in PBS solution (pH 7.4) at 37 °C in static conditions. The water absorption after 42 days was approximately 1.5%, regardless of the inclusion of the G1 or G3 dendrimers (Figure 5a). In all cases, the majority of the water absorption was achieved during the first 7 days with only incremental increases until day 42. Moreover, a degradability testing was conducted after 56 days following similar conditions to the water absorption experiments (Figure 5b). The mass loss of the cross-linked composites averaged approximately 0.2 wt %. More specifically, the mass loss of the BC, 5 wt % G1 and 5 wt % G3 were 0.189 (0.007), 0.269 (0.011), and 0.170 (0.010) wt %, respectively. Again, it was surprising that the mass loss of the 5 wt % G3 was the lowest considering it contained the highest density of hydrolyzable ester bonds (0.297 mmol/g, as compared to 0.208 mmol/g for the 5 wt % G1, and 0 mmol/g for the BC). The higher mass loss for the 5 wt % G1 composite could potentially be attributed to better compatibilization during the mixing procedure, due to lower viscosity, and therefore better distribution in the cross-linked network when compared to the larger G3 dendrimer. These results were in good agreement with the widely used biodegradable polycarprolactone (PCL) with mass loss lower than 0.2 wt % after 8 weeks at 37 °C under PBS (pH = 7.4).^40^ Overall, the introduction of low- and high-generation dendrimers, in concentrations up to 5 wt %, was found to yield curable composites with promising mechanical properties.

Cytotoxicity was assessed using the Alamar Blue assay in both Raw 264.7 and HaCaT cells. In this experiment, the BC material was studied as a reference alongside 5 wt % G1 and G3 composites. According to the ISO10993–5:200947 standard, materials exhibiting less than 70% cell viability are considered potentially cytotoxic.^41^ As represented in Figure 5c, the treatment with all composites in both cell lines provided results of cell viability higher than 85%, placing these materials as promising candidates with excellent cytotoxic profiles ready for further evaluation as composites for biomedical applications.

From an overall structure-to-property perspective, the introduction of dendritic additives to the TATO composite influences the processing parameter while maintaining adequate mechanical performance. The addition of 5 wt % of the dendrimers, with branched dendritic configuration, substantially alters the viscosity of the resin formulations. This is indeed fascinating considering that the G1 and G3 dendrimers of molecular weight 723 and 3534 g/mol, respectively, are significantly larger molecules than the allyl functional TATATO monomer with a molecular weight of 249 g/mol. Additionally, lower generation polyester dendrimers (G1) in 3 or 5 wt % yield narrower size distribution of pores in comparison to larger dendrimers (G3) or the neat composites. This could be correlated to the decreased formulation viscosity and increased molecular mobility that counteract the formation of larger air bubbles. The degradation assessment of the composites also suggests that lower generation dendrimers (G1) accelerate the degradation of the TATO composites when compared with composites with larger dendrimers (G3) or the BC.

## 4 Conclusions

Strong TATO composites based on 1,3,5-triazine-2,4,6-trione, 1,3,5-tris(3-mercaptopropyl) triazine-2,4,6-trione, and hydroxyapatite are considered as a potential platform that can be implemented as an alternative solution to metal plates during the fixation of bone fractures. To further expand on this library of exciting composites, allyl-functionalized polyester dendrimers based on bis-MPA of generations 1 and 3 were introduced as branched cross-linking additives and their impact was compared to BC with a flexural modulus above 7 GPa and flexural strength of 61 MPa. The addition of 1, 3, and 5 wt % of the G1 or G3 dendrimers to the TATO composite formulation decreased the mixture viscosity and even reached Newtonic, nonshear thinning properties for the formulation with 5 wt % of G1 dendrimer. All formulations were efficiently cross-linked via HEV-TEC and the produced composites displayed an overall decreased *T*_g_ with flexural modulus above 6 GPa and flexural strength up to 51 MPa when measured in dry conditions. Furthermore, the composites with the highest wt % of dendrimers as additives and ester bond density, 5 wt % G1 and G3, reached a maximum water absorption of ca. 1.5%, mass loss up to 0.27%, which is in the range of established biodegradable PCL, and with promising cytotoxicity profiles. Indeed, polyester dendrimers, of generation 1 and 3, provide promising features to hydroxyapatite-infused TATO systems, both during mixing as well as postcuring. The decreased viscosity profile of the formulations coupled with the maintained high modulus of the cured TATO composites, upon the addition of dendrimers as multifunctional cross-linking additives, widen the scope toward a new generation of composites with higher HA content and therefore stronger materials. These systems will be further optimized and investigated as potential bone fracture fixation patches including animal studies.

## Data Availability

Research data are not shared.

## Supporting Information Available

Supporting Information including supplementary data of the formulations, Raman spectrum, FE-SEM images, and viscosities (PDF)
