# Supplementary material for: Impact of Polyester Dendrimers as Branched Multifunctional Cross-Linking Additives in Triazine-Trione-Based Composites Developed via High-Energy Visible Light Thiol–ene Chemistry
Source: ACS Appl Polym Mater. 2023 Nov 27;5(12):10395–403. doi: 10.1021/acsapm.3c02246 (PMC10714353; doi:10.1021/acsapm.3c02246)
Supplement: Supplementary file 1 — ap3c02246_si_001.pdf [file ap3c02246_si_001.pdf]

Supporting information

The impact of polyester dendrimers as branched multifunctional crosslinking additives in triazine-trione based composites developed via high-energy visible light thiol-ene chemistry

*Jinjian Lin, Natalia Sanz del Olmo, Jorge San Jacinto Garcia, Faridah Namata, Daniel J. Hutchinson, and Michael Malkoch**

KTH Royal Institute of Technology, School of Engineering Sciences in Chemistry, Biotechnology and Health (CBH), Department of Fibre and Polymer Technology, Teknikringen 56-58, SE-100 44 Stockholm, Sweden

*E-mail: malkoch@kth.se

Table S1. The formulations for different composites investigated in this study

Materials	Dendrimers and concentrations	m (TATATO) (mg)	m (TMTATO) (mg)	m (Cat57) (mg)	m (Dendrimers) (mg)	m (TPO) (mg)	M (56wt% HA) (mg)
**BC**	No dendrimer included	1375.69	2000	77.35	-	19.67	4419.89
**1wt% G1**	**G1 Dendrimer** (1wt%)	1321.65	2000	74.31	79.62	18.90	4506.36
**1wt% G3**	**G3 Dendrimer** (1wt%)	1331.55	2000	74.87	80.21	19.04	4518.72
**3wt% G1**	**G1 Dendrimer** (3wt%)	1212.27	2000	68.16	243.43	17.33	4561.83
**3wt% G3**	**G3 Dendrimer** (3wt%)	1241.28	2000	69.79	245.26	17.75	4607.18
**5wt% G1**	**G1 Dendrimer** (5wt%)	1101.28	2000	61.92	409.55	15.75	4599.73
**5wt% G3**	**G3 Dendrimer** (5wt%)	1146.94	2000	64.49	417.32	16.40	4698.29

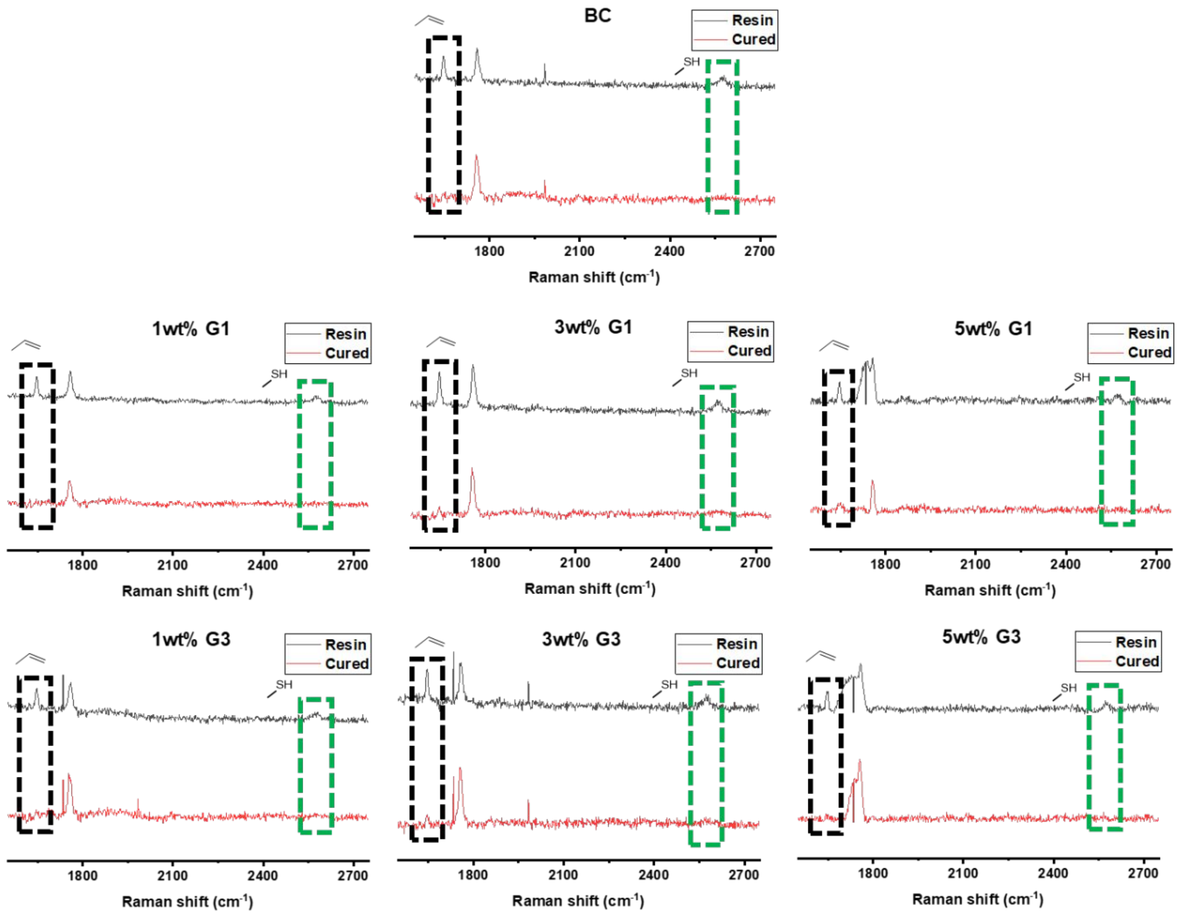

Figure S1. Raman spectrum for all the formulations before and after HEV light fast curing. The full TEC monomers conversion is confirmed by the disappearance of raman shifts at 1630-1660 cm^{-1} (C-C double bond) and 2560-2600 cm^{-1} (-SH).

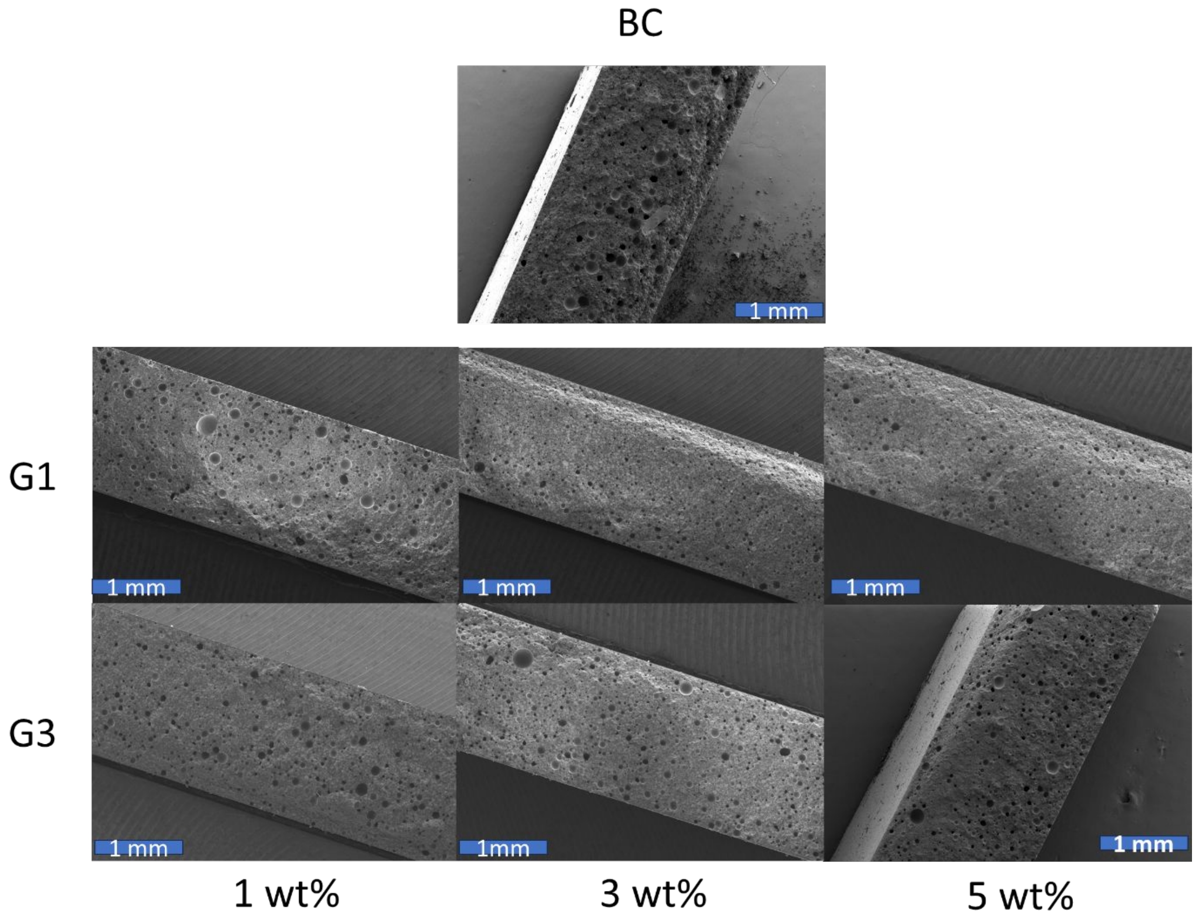

Figure S2. SEM images of the cross-sections for all the composites *i.e.* BC, 1wt% G1, 1wt% G3, 3wt% G1, 3wt% G3, 5wt% G1 and 5wt% G3, respectively. The scale of measurement is 1 mm.

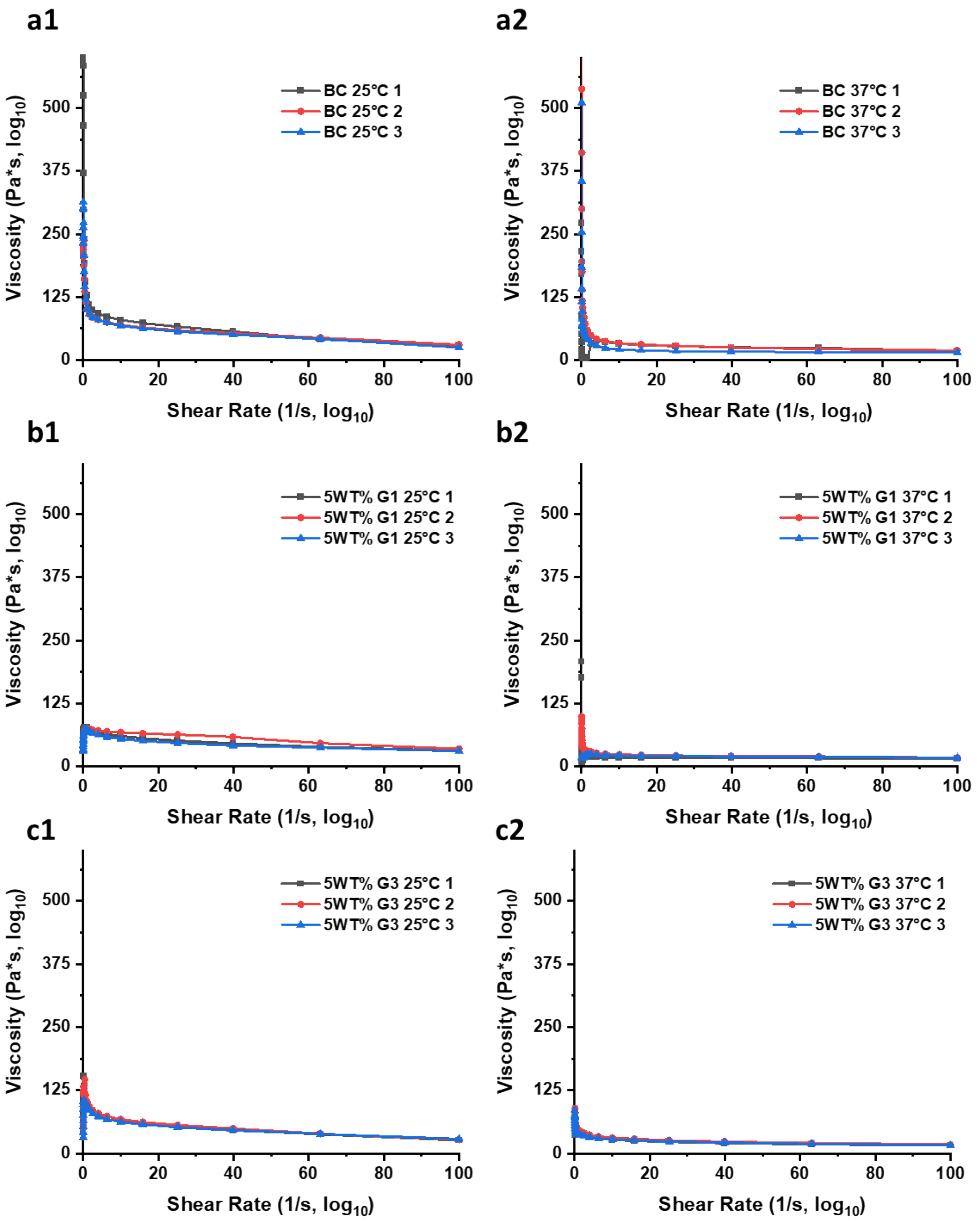
a1
BC 25°C 1
BC 25°C 2
BC 25°C 3
Viscosity (Pa*s, log_{10})
Shear Rate (1/s, log_{10})
a2
BC 37°C 1
BC 37°C 2
BC 37°C 3
Viscosity (Pa*s, log_{10})
Shear Rate (1/s, log_{10})
b1
5WT% G1 25°C 1
5WT% G1 25°C 2
5WT% G1 25°C 3
Viscosity (Pa*s, log_{10})
Shear Rate (1/s, log_{10})
b2
5WT% G1 37°C 1
5WT% G1 37°C 2
5WT% G1 37°C 3
Viscosity (Pa*s, log_{10})
Shear Rate (1/s, log_{10})
c1
5WT% G3 25°C 1
5WT% G3 25°C 2
5WT% G3 25°C 3
Viscosity (Pa*s, log_{10})
Shear Rate (1/s, log_{10})
c2
5WT% G3 37°C 1
5WT% G3 37°C 2
5WT% G3 37°C 3
Viscosity (Pa*s, log_{10})
Shear Rate (1/s, log_{10})

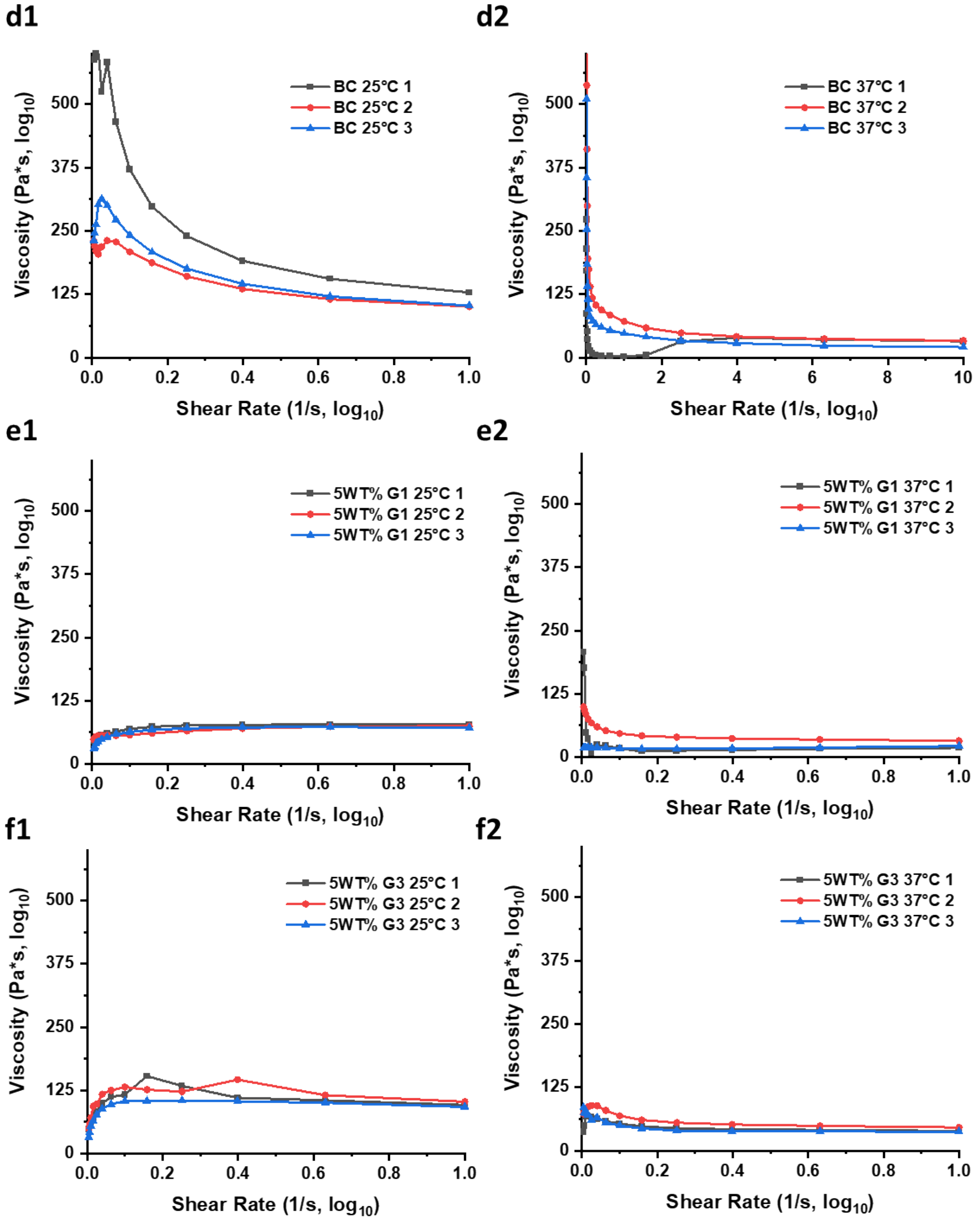
d1
Viscosity (Pa*s, log10)
0
125
250
375
500
Shear Rate (1/s, log10)
0.0
0.2
0.4
0.6
0.8
1.0
BC 25°C 1
BC 25°C 2
BC 25°C 3
d2
Viscosity (Pa*s, log10)
Shear Rate (1/s, log10)
0
2
4
6
8
10
BC 37°C 1
BC 37°C 2
BC 37°C 3
e1
Viscosity (Pa*s, log10)
Shear Rate (1/s, log10)
5WT% G1 25°C 1
5WT% G1 25°C 2
5WT% G1 25°C 3
e2
Viscosity (Pa*s, log10)
Shear Rate (1/s, log10)
5WT% G1 37°C 1
5WT% G1 37°C 2
5WT% G1 37°C 3
f1
Viscosity (Pa*s, log10)
Shear Rate (1/s, log10)
5WT% G3 25°C 1
5WT% G3 25°C 2
5WT% G3 25°C 3
f2
Viscosity (Pa*s, log10)
Shear Rate (1/s, log10)
5WT% G3 37°C 1
5WT% G3 37°C 2
5WT% G3 37°C 3

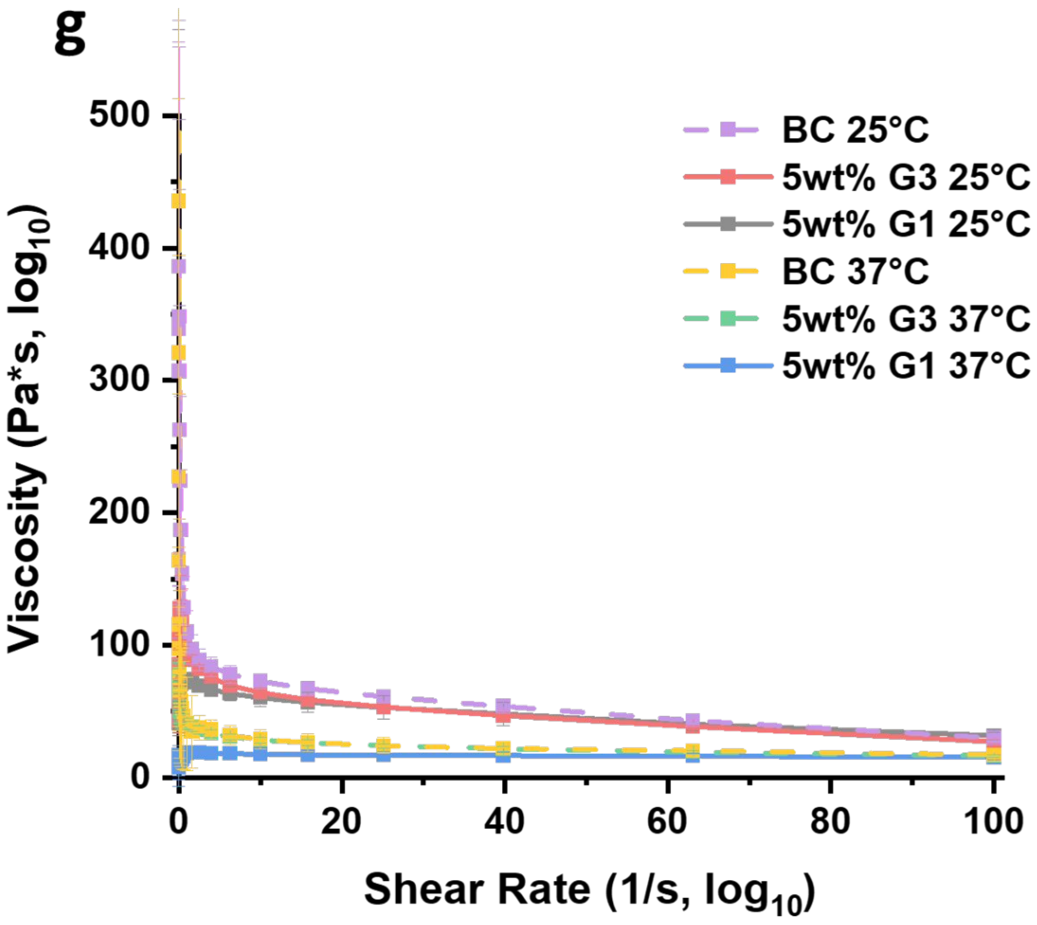

Figure S3. Viscosity versus shear rate for the BC, 5wt% G1 and 5wt% G3 composites under both 25 °C and 37 °C. (a-c) shear rate = $10^{-4} – 10^{2}$ 1/s, $\log_{10}$, (d-f) shear rate = $10^{-4} – 1$ 1/s, $\log_{10}$. (g) compilations of averaging viscosity, for each material under both 25 °C and 37 °C, versus shear rate scale ($10^{-4} – 10^{2}$ 1/s, $\log_{10}$) with standard deviations.
